# Preoperative prediction model for macrotrabecular-massive hepatocellular carcinoma based on contrast-enhanced CT and clinical characteristics: a retrospective study

**DOI:** 10.3389/fonc.2023.1124069

**Published:** 2023-05-01

**Authors:** Chutong He, Wanli Zhang, Yue Zhao, Jiamin Li, Ye Wang, Wang Yao, Nianhua Wang, Wenshuang Ding, Xinhua Wei, Ruimeng Yang, Xinqing Jiang

**Affiliations:** ^1^Department of Radiology, The Second Affiliated Hospital, School of Medicine, South China University of Technology, Guangzhou, Guangdong, China; ^2^Department of Radiology, Central People’s Hospital of Zhanjiang, Zhanjiang, Guangdong, China; ^3^Department of Pathology, The Second Affiliated Hospital, School of Medicine, South China University of Technology, Guangzhou, Guangdong, China

**Keywords:** hepatocellular carcinoma, liver neoplasm, computed tomography, macrotrabecular-massive, diagnosis

## Abstract

**Objective:**

To investigate the predictive value of contrast-enhanced computed tomography (CECT) imaging features and clinical factors in identifying the macrotrabecular-massive (MTM) subtype of hepatocellular carcinoma (HCC) preoperatively.

**Methods:**

This retrospective study included 101 consecutive patients with pathology-proven HCC (35 MTM subtype *vs*. 66 non-MTM subtype) who underwent liver surgery and preoperative CECT scans from January 2017 to November 2021. The imaging features were evaluated by two board-certified abdominal radiologists independently. The clinical characteristics and imaging findings were compared between the MTM and non-MTM subtypes. Univariate and multivariate logistic regression analyses were performed to investigate the association of clinical-radiological variables and MTM-HCCs and develop a predictive model. Subgroup analysis was also performed in BCLC 0-A stage patients. Receiver operating characteristic (ROC) curves analysis was used to determine the optimal cutoff values and the area under the curve (AUC) was employed to evaluate predictive performance.

**Results:**

Intratumor hypoenhancement (odds ratio [OR] = 2.724; 95% confidence interval [CI]: 1.033, 7.467; *p* = .045), tumors without enhancing capsules (OR = 3.274; 95% CI: 1.209, 9.755; *p* = .03), high serum alpha-fetoprotein (AFP) (≥ 228 ng/mL, OR = 4.101; 95% CI: 1.523, 11.722; *p* = .006) and high hemoglobin (≥ 130.5 g/L; OR = 3.943; 95% CI: 1.466, 11.710; *p* = .009) were independent predictors for MTM-HCCs. The clinical-radiologic (CR) model showed the best predictive performance, achieving an AUC of 0.793, sensitivity of 62.9% and specificity of 81.8%. The CR model also effectively identify MTM-HCCs in early-stage (BCLC 0-A stage) patients.

**Conclusion:**

Combining CECT imaging features and clinical characteristics is an effective method for preoperatively identifying MTM-HCCs, even in early-stage patients. The CR model has high predictive performance and could potentially help guide decision-making regarding aggressive therapies in MTM-HCC patients.

## 1 Introduction

Hepatocellular carcinoma (HCC) is the third leading cause of cancer-related deaths worldwide (1), and the prognosis remains poor, with a high recurrence rate of up to 80% within five years after surgical resection (2). Recently, a newly classified variant histological HCC subtype, namely macrotrabecular-massive HCC (MTM-HCC), was reported in 2019 WHO classification of digestive system tumors (3). Due to the gene and molecular-related proliferative activity (e.g., *TP53* mutations, *FGF19* amplification, and chromosomal instability), MTM-HCC represents an aggressive form of HCC that is associated with poor clinical prognosis, especially early recurrence (4–6). MTM-HCC is a promising candidate subtype for immunotherapy (7), which further implies the potential significance of identifying this HCC subtype for tailored clinical management.

As the gold standard for MTM-HCC diagnosis, pathological examinations (including surgical resection or biopsy) have limitations, such as invasiveness, complications, and sampling errors. Additionally, HCC is unique since it can be diagnosed by the typical radiologic features in high-risk patients according to the current HCC clinical guidelines, so histological evaluation is not mandatory for diagnosis (2, 8, 9). Thus, developing a noninvasive, robust method to preoperatively predict MTM-HCC is urgently needed. Recently, ultrasound (US), computed tomography (CT), and magnetic resonance image (MRI) have been applied for identifying MTM-HCC based on specific imaging features (10–16), particularly Gd-EOB-DTPA enhanced MRI (10, 11, 13, 15). Compared to MRI, contrast-enhanced CT (CECT) has the advantages of fast speed and low cost and is comparable to MRI for MTM-HCC prediction when using similar diagnostic criteria as MRI (16). However, the value of CECT in differentiating MTM from non-MTM HCC has not been fully determined.

In addition, most previous studies focused on imaging features without fully assessing the contribution of clinical factors to predictive performance. Clinical characteristics such as biochemical and tumor biomarkers also play an important role in HCC diagnosis and prognosis (17–22), with the MTM-HCC being associated with a higher Barcelona Clinical Liver Cancer (BCLC) stage, poor histologic differentiation and higher serum alpha-fetoprotein (AFP) (6, 23). Neutrophil-lymphocyte-ratio (NLR), platelet-lymphocyte-ratio (PLR), γ-glutamyl transpeptidase-lymphocyte ratio (GLR), and other lab results are also related to poor prognosis and therapeutic effects in HCC (17, 19, 20, 22). However, the contribution of clinical characteristics in identifying MTM-HCCs remains unclear. Therefore, this study investigated whether CECT-based image features or/and clinical data-derived predictive models could help preoperatively identify MTM-HCCs.

## 2 Materials and methods

### 2.1 Study participants

**Figure 1:**
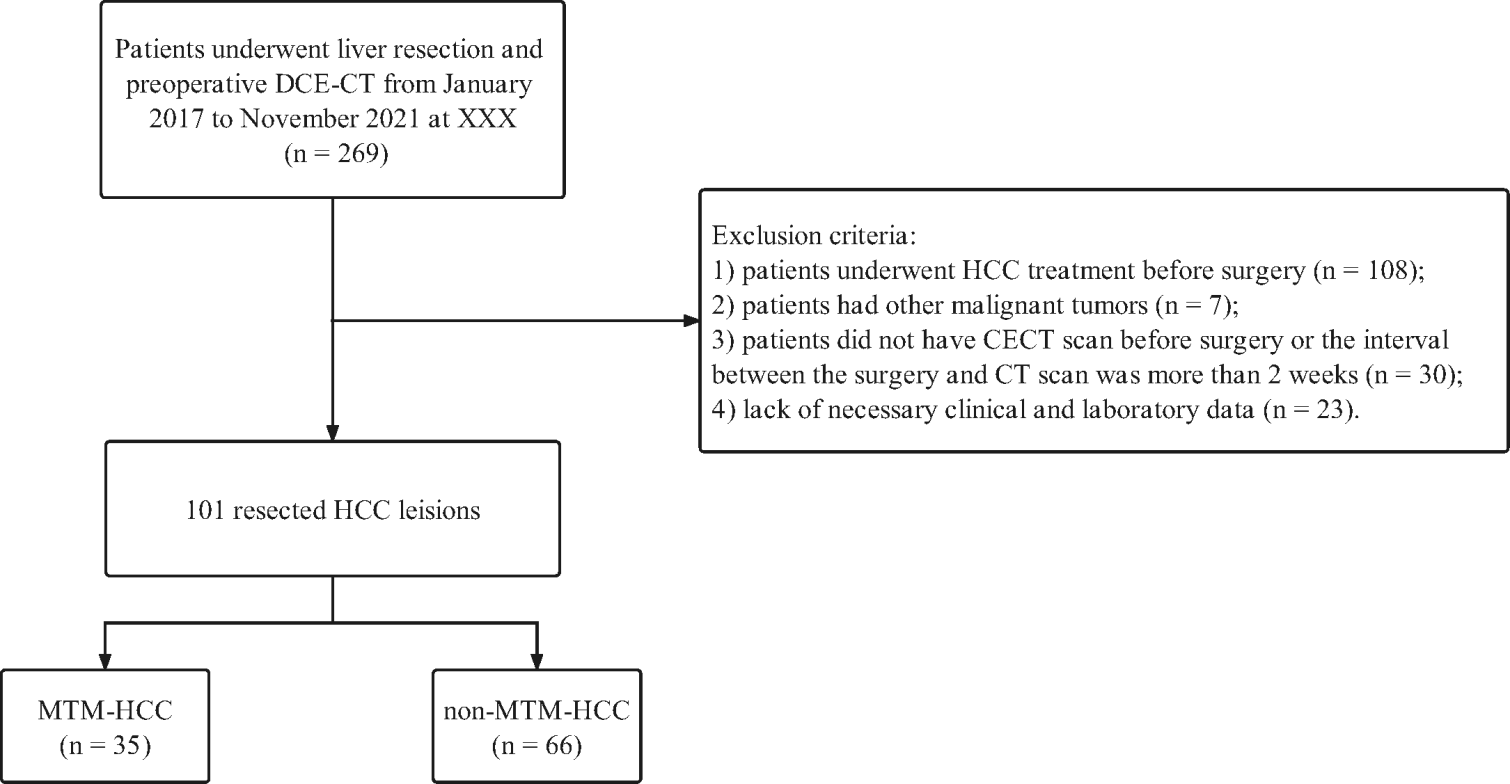
Flowchart of this retrospective study.

This retrospective study was approved by the Institutional Review Board of the Second Affiliated Hospital of South China University of Technology, and written informed consent was waived. The study protocol conforms to the ethical guidelines of the 1975 Declaration of Helsinki as reflected in *a priori* approval by the institution’s human research committee. Consecutive patients who underwent liver surgery and had postoperative pathological proven HCC from January 2017 to November 2021 were recruited. The exclusion criteria were 1) patients who underwent HCC treatment before surgery (n = 108); 2) patients who had other malignant tumors (n = 7); 3) patients who did not have a CECT scan before surgery or the interval between the surgery and CT scan was more than two weeks (n = 30); 4) lack of necessary clinical and laboratory data (n = 23). The study flowchart is displayed in **Figure 1**.

The basic demographic and clinical characteristics, including gender, age, background liver diseases, serum tumor biomarkers, liver function tests, and some common laboratory tests, were obtained from the clinical data system. The clinical characteristics were calculated according to the following formulae, and the ALBI values were categorized according to a previous study (22):

NLR = Neutrophils counts/Lymphocyte counts (1)
PLR = Platelet counts/Lymphocyte counts (2)
GLR = γ-Glutamyl transpeptidase/Lymphocyte counts (3)
ALBI = 0.66 × lg (TBIL) – 0.085 × ALB (4)

### 2.2 Pathological analysis

All histological slides were reviewed by a pathologist (W.S. Ding) with 16 years of experience blinded to the other clinical and imaging results. MTM-HCC was defined as tumors with a predominant (> 50%) macrotrabecular architecture pattern (trabeculae more than six cells thick) according to previously reported diagnostic criteria (6). Tumor differentiation was evaluated by the Edmondson-Steiner grading system, and microvascular invasion (MVI) and the Ki-67 index were also evaluated.

### 2.3 CT scanning protocols

All dynamic acquisition of contrast-enhanced CT images were obtained prospectively using the following CT scanners: Siemens Somatom Drive, Siemens Somatom Force, Philips Brilliance 64, and Toshiba Aquilion One. The scanning parameters were as follows: tube voltage, 120 kVp; tube current, 200 mAs; reconstruction slice thickness, 5-7 mm. After unenhanced images were acquired, all patients were injected with intravenous nonionic iodinated contrast agent (iodipamide, 370 mg I/mL, Bracco) *via* the antecubital vein by mechanical power injectors based on their weight (2.0 mL/kg body weight, maximal dose of 180 mL), followed by a 20 mL saline flush. Four phases of contrast-enhanced CT (early arterial phase, late arterial phase, portal venous phase, and delayed phase) were obtained in 18-25 s, 35-40 s, 50-60 s, and 120-250 s, respectively, after contrast injection.

### 2.4 Evaluation of CT imaging features

The CT images were reviewed independently by two radiologists (C.T. He and W.L. Zhang, with 3 and 5 years of experience in abdominal diagnostic imaging, respectively), who were blinded to the detailed clinical and pathological results. All image features were assessed according to the Liver Imaging Reporting and Data System (LI-RADS) version 2018 (24) and previous studies, including (*a*) LI-RADS major features, (*b*) LI-RADS ancillary features, and (*c*) non-LI-RADS features according to previous studies.

- LI-RADS major features included *i*) non-rim arterial phase hyperenhancement (APHE), defined as a lesion with an arterial phase attenuation higher than the background liver without rim enhancement; *ii*) washout, defined as non-peripheral relative hypoattenuation of the lesion compared with background liver on the portal venous and delayed phases; and *iii*) enhancing capsule, defined as a smooth, uniform, sharp border around the tumor at the portal venous phase or delayed phase as an enhancing rim.
- LI-RADS ancillary features included *i*) peritumoral hyperenhancement, defined as hyperenhancement adjacent to the tumor at the late arterial phase or portal venous phase; *ii*) non-smooth tumor margins, non-smooth tumor margins were assessed according to the morphologic appearance of the tumor at the delayed phase; *iii*) mosaic architecture, defined as a lesion with multiple compartments of different density and enhancement, separated by septations; *iv*) blood products in mass, intralesional or perilesional hemorrhage in the absence of biopsy or trauma; *v*) fat in mass, defined as the presence of macroscopic fat within the tumor; and *vi*) tumor in vein, defined as the presence of enhancing soft tissue in the portal venous and/or inferior vena cava, regardless of visualization of a parenchymal mass.
- non-LI-RADS features included *i*) intratumor hypoenhancement and intratumor artery. Intratumor hypoenhancement was defined as lesion components without or mild enhancement (with lower attenuation than the adjacent normal liver parenchyma at the arterial phase) accounts for more than 20% of the whole tumor volume, including necrosis and severe ischemia. While *ii*) intratumor artery was defined as the presence of hepatic artery within the tumor.

The long and short axes of each tumor section were measured with the largest tumor diameter at the portal venous phase. Discrepancies were solved by consulting with a senior radiologist (R.M. Yang) with 15 years of experience in abdominal diagnostic radiology.

### 2.5 Model construction

The prediction model was constructed using univariate and multivariate logistic regression. Variables with *p* <.10 in the univariate logistic regression analysis were included in the backward stepwise multivariate logistic regression. Three models were developed: (*i*) a model based on clinical characteristics (C model), (*ii*) a model based on radiological semantic features (R model), and (*iii*) a model combining both clinical characteristics and radiological semantic features (CR model). To dichotomize the continuous variables, receiver operating characteristic (ROC) curves were employed to determine the threshold by calculating the maximum Youden index.

### 2.6 Subgroup analysis

Although MTM-HCCs appeared more frequent in higher BCLC stage, patients in BCLC 0-A stage might achieve more aggressive therapies earlier if their tumors presented as MTM-HCCs. The performance of the three models in BCLC 0-A stage were then evaluated.

### 2.7 Statistical analysis

Continuous variables are reported as means and standard deviations (SD) or medians and interquartiles. The categorical variables are presented as numbers and proportions. The normality of data distribution was assessed by the Shapiro-Wilk test. Comparisons between groups were performed using the following statistical tests: Student’s *t*-tests for normally distributed continuous variables, the Mann-Whitney *U* test for non-normally distributed continuous variables, and the Chi-square test for binary categorical variables. Receiver operating characteristic (ROC) curves were employed to determine the threshold of the continuous variables by calculating the maximum Youden index. Features with *p* <.10 in the univariate logistic regression analysis were then included in backward stepwise multivariate logistic regression to develop MTM-HCC predictive models. ROC curve and area under the curve (AUC) were then applied to evaluate the performance of the constructed models, followed by DeLong’s test to compare the AUC value between the two models. All statistical analyses were performed using the SPSS software (version 23.0, IBM Corp.) and R software (version 4.1.3, http://www.r-project.org). A *p*-value <.05 was considered statistically significant.

## 3 Results

### 3.1 Clinical and pathological characteristics

A total of 101 patients (80 males and 21 females) were included in this study, including 35 MTM-HCCs and 66 non-MTM-HCCs. The baseline characteristics, demographic and pathological data are summarized in **Table 1**. The mean age of the study cohort was 58.03 ± 13.91 years old (ranging from 24 to 86 years old). Among the 101 patients, 68 patients (67.3%) were HBV infected, 48 patients (47.5%) presented with microvascular invasion (MVI), and 49 patients (48.5%) had a Ki-67 index higher than 10%. The clinical characteristics are summarized in **Table 2**. MTM-HCC patients had a higher BCLC stage (*p* = .02), higher serum AFP levels (*p* = .02), and higher hemoglobin levels (*p* = .02).

**Table 1:** Baseline demographic, clinical, and pathologic characteristics of MTM-HCCs and non-MTM-HCCs.

	Total cohort (n = 101)	MTM-HCC (n = 35)	non-MTM-HCC (n = 66)	*p*-value
Sex				.89
Male	80 (79.2%)	28 (80.0%)	52 (78.8%)	
Female	21 (20.8%)	7 (20.0%)	14 (21.2%)	
Age (years)*	58.03 ± 13.91	54.94 ± 14.41	59.67 ± 13.46	.11
Child-Pugh				.24
A	92 (91.1%)	34 (97.1%)	58 (87.9%)	
B	9 (8.9%)	1 (2.9%)	8 (12.1%)	
HBV infection				.13
Yes	68 (67.3%)	27 (77.1%)	41 (62.1%)	
No	33 (32.7%)	8 (22.9%)	25 (37.9%)	
BCLC stage				**.02**
0-A	82 (81.2%)	24 (68.6%)	58 (87.9%)	
B-C	19 (18.8%)	11 (31.4%)	8 (12.1%)	
Cirrhosis				.10
Yes	43 (42.6%)	11 (31.4%)	32 (48.5%)	
No	58 (57.4%)	24 (68.6%)	34 (51.5%)	
Tumor location				.34
Left lobe	26 (25.7%)	7 (20.0%)	19 (28.8%)	
Right lobe	75 (74.3%)	28 (80.0%)	47 (71.2%)	
E-S grade				.39
I-II	43 (42.6%)	13 (37.1%)	30 (46.2%)	
III-IV	57 (57.4%)	22 (62.9%)	35 (53.8%)	
MVI status				.09
Positive	49 (48.5%)	21 (60.0%)	28 (42.4%)	
Negative	52 (51.5%)	14 (40.0%)	38 (57.6%)	
Ki-67 index				.99
≤ 10%	52 (51.5%)	18 (51.4%)	34 (51.5%)	
> 10%	49 (48.5%)	17 (48.6%)	32 (48.5%)	
Size (mm)^†^	54.00 [35.50, 77.00]	59.00 [40.00, 90.00]	53.00 [33.50, 73.00]	.14

MTM-HCC, macrotrabecular-massive hepatocellular carcinoma; E-S, Edmondson-Steiner; BCLC, Barcelona Clinical Liver Cancer; MVI, microvascular invasion.

Data are presented as actual numbers and frequencies in parentheses unless otherwise stated.

^*^Data are presented as mean values ± standard deviation (SD).

^†^Data are presented as median values and interquartile.

Significant values (p <.05) are presented in bold.

**Table 2:** Comparison of the laboratory characteristics of MTM-HCCs and non-MTM-HCCs.

	MTM-HCC (n = 35)	non-MTM-HCC (n = 66)	*p*-value
WBC (10^9^/L)	6.78 ± 1.94	7.00 ± 2.68	.66
RBC (10^12^/L)	4.72 ± 0.75	4.43 ± 0.84	.09
PLT (10^9^/L)	236.49 ± 76.24	208.18 ± 79.83	.09
HGB (g/L)	140.29 ± 18.99	130.24 ± 21.53	***.02***
N (10^9^/L)	4.20 ± 1.56	4.40 ± 2.50	.66
L (10^9^/L)	1.73 ± 0.76	1.61 ± 0.59	.38
ALT (U/L)	46.60 ± 38.99	41.53 ± 32.75	.58
AST (U/L)	55.37 ± 36.93	44.50 ± 27.92	.10
GGT (U/L)	118.97 ± 108.83	116.03 ± 144.60	.92
TBIL (μmol/L)	16.21 ± 6.05	17.27 ± 9.45	.92
DBIL (μmol/L)	4.73 ± 2.58	5.60 ± 5.68	.39
ALB (g/L)	38.09 ± 4.17	37.25 ± 2.77	.38
PT (s)	13.21 ± 0.95	13.32 ± 1.02	.61
FIB (g/L)	3.54 ± 0.95	3.45 ± 1.37	.71
AFP (ng/mL)	257.00 [4.65, 5943]	12.53 [3.30, 181.25]	***.02***
CEA (ng/mL)	3.06 ± 2.06	2.93 ± 1.65	.74
NLR	3.20 ± 3.15	3.11 ± 2.59	.38
PLR	179.44 ± 127.71	142.29 ± 70.60	.16
GLR	82.80 ± 78.19	82.42 ± 109.77	.99
GGT/AST	2.28 ± 1.79	2.81 ± 3.06	.35
AST/ALT	1.45 ± 0.81	1.34 ± 0.88	.51
ALB/FIB	11.71 ± 3.92	12.32 ± 4.66	.51
PLT/WBC	36.02 ± 10.75	32.34 ± 13.88	.18
ALBI grade^‡^			.32
I	13 (37.1%)	17 (25.8%)	
II	22 (62.9%)	47 (71.2%)	
III	0 (0.0%)	2 (3.0%)	

MTM-HCC, macrotrabecular-massive hepatocellular carcinoma; WBC, white blood cell; RBC, red blood cell; PLT, platelet; HGB, hemoglobin; N, neutrophil; L, lymphocyte; ALT, alanine aminotransferase; AST, aspartate aminotransferase; GGT, γ-glutamyl transpeptidase; TBIL, total bilirubin; DBIL, direct bilirubin; ALB, albumin; PT, prothrombin time; FIB, fibrinogen; AFP, alpha-fetoprotein; CEA, carcinoembryonic antigen; NLR, neutrophil-lymphocyte-ratio; PLR, platelet-lymphocyte-ratio; GLR, γ-glutamyl transpeptidase-lymphocyte-ratio; GGT/AST, γ-glutamyl transpeptidase-aspartate aminotransferase-ratio; AST/ALT, aspartate aminotransferase-alanine aminotransferase-ratio; ALB/FIB, albumin-fibrinogen-ratio; PLT/WBC, platelet-white blood cell ratio; ALBI, albumin-bilirubin grade.

Data are presented as mean values ± standard deviation (SD) unless otherwise stated.

^†^Data are presented as median values and interquartile.

^‡^Data are presented as actual amounts and frequencies in parentheses.

Significant values (p <.05) are presented in bold.

### 3.2 Imaging findings

**Figure 2:**
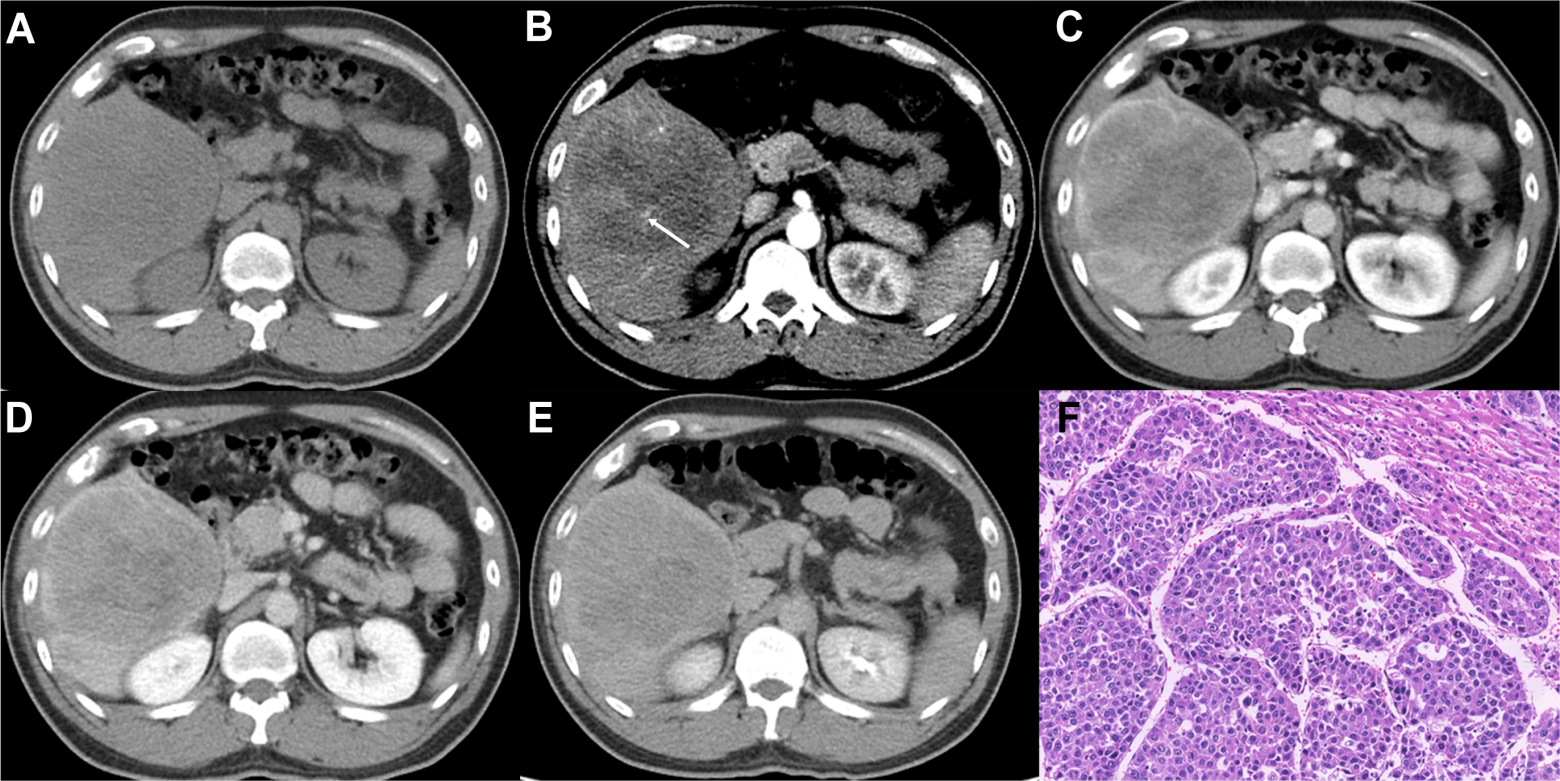
Imaging features and pathological information of a 37-year-old male MTM-HCC patient with an AFP level of 245,658 ng/mL and hemoglobin of 176 g/L. Multiphase axial contrast-enhanced CT (CECT) displays low attenuation in the unenhanced phase **(A)**, the intratumor artery (white arrow) in the early arterial phase **(B)**, rim hyperenhancement (APHE) and intratumor hypoenhancement in the late arterial phase **(C)**, portal venous phase **(D)**, and delay phase **(E)**. Histopathology revealed the predominant thick trabecular structure diagnosed as MTM-HCC (**F**, original magnification, × 100; hematoxylin-eosin staining).

**Figure 3:**
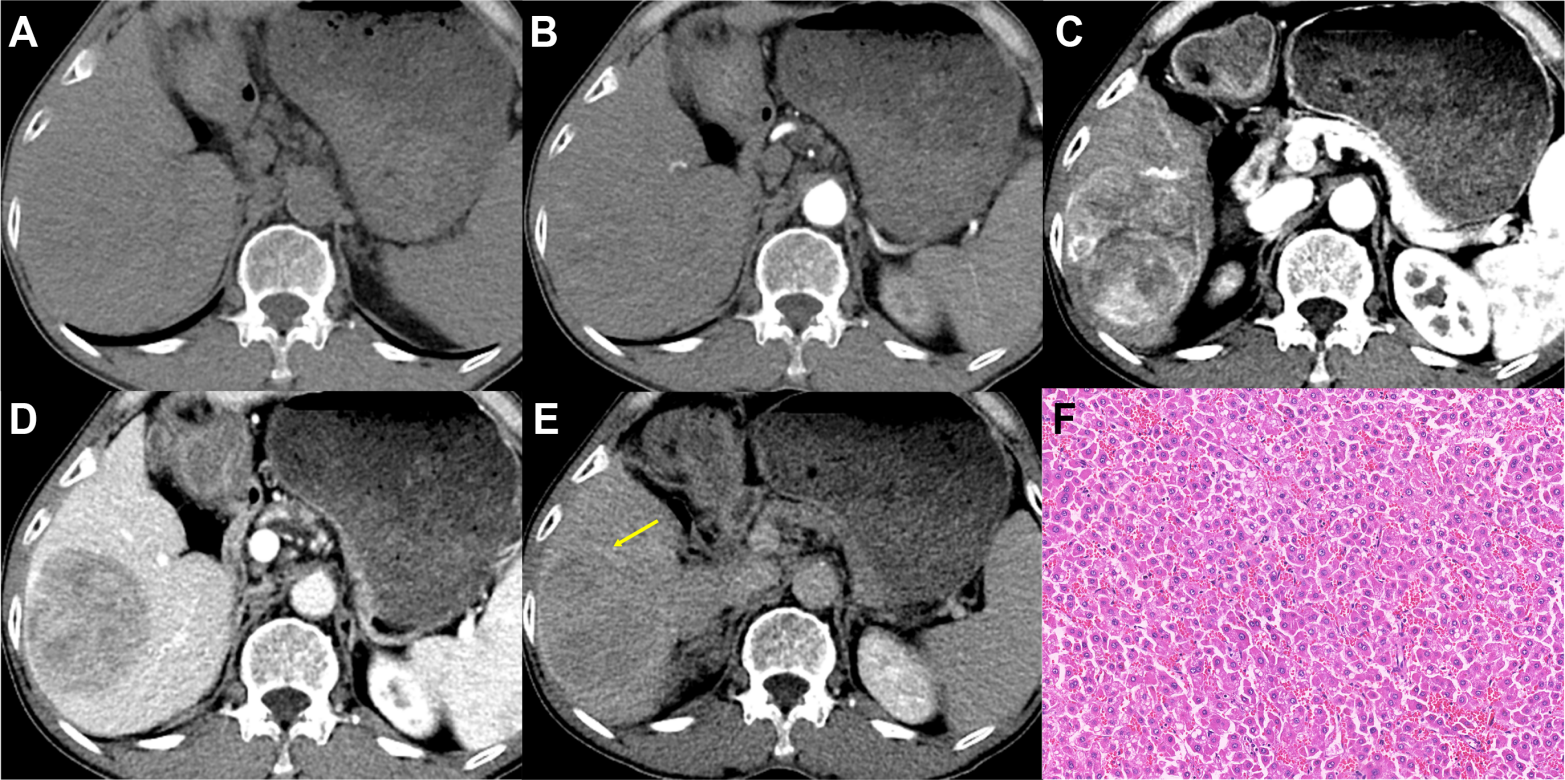
Imaging features and pathological information of a 62-year-old male non-MTM-HCC patient with an AFP level of 26.58 ng/mL and hemoglobin of 119 g/L. Multiphase axial contrast-enhanced CT (CECT) demonstrates iso-attenuation in the unenhanced phase **(A)** and early arterial phase **(B)**. Strong and heterogeneous hyperenhancement (APHE) and little intratumor hypoenhancement are evident in the late arterial phase **(C)**. Capsule enhancement (yellow arrow) and washout are demonstrated in the portal venous phase **(D)** and delay phase **(E)**. Histopathology confirmed the diagnosis of non-MTM-HCC (**F**, original magnification, × 100; hematoxylin-eosin staining).

**Table 3** summarizes the imaging features of MTM-HCCs and non-MTM-HCCs, showing that MTM-HCCs had a higher probability of more than 20% intratumor hypoenhancement (*p* = .01). The other features were similar in both groups and representative images of MTM-HCCs and non-MTM-HCCs are illustrated in **Figures 2**, **3**.

**Table 3:** Comparison of the CT imaging features of MTM-HCCs and non-MTM-HCCs.

	MTM-HCC (n = 35)	non-MTM-HCC (n = 66)	*p*-value
Non-rim APHE	32 (91.4%)	61 (92.4%)	> 0.99
Washout	31 (88.6%)	57 (86.4%)	> 0.99
Enhancing capsule	12 (34.3%)	35 (53.0%)	.07
Peritumoral hyperenhancement	10 (28.6%)	9 (13.6%)	.07
Non-smooth tumor margin	31 (88.6%)	51 (77.3%)	.27
Mosaic architecture	23 (65.7%)	41 (62.1%)	.72
Blood products in mass	1 (2.9%)	4 (6.1%)	.66
Fat in mass	1 (2.9%)	1 (1.5%)	> 0.99
Intratumor hypoenhancement > 20%	20 (57.1%)	21 (31.8%)	***.01***
Internal artery	20 (57.1%)	37 (56.1%)	.92
Tumor in vein	7 (20.0%)	7 (10.6%)	.19
Long-to-short-axis ratio^*^	1.01 ± 0.20	1.03 ± 0.20	.71

MTM-HCC, macrotrabecular-massive hepatocellular carcinoma; APHE, arterial phase hyperenhancement.

Data are presented as numbers and frequencies in parentheses unless otherwise stated.

^*^Data are presented as mean values and standard deviation.

Significant values (p <.05) are presented in bold.

### 3.3 Predictors of MTM-HCCs

**Figure 4:**
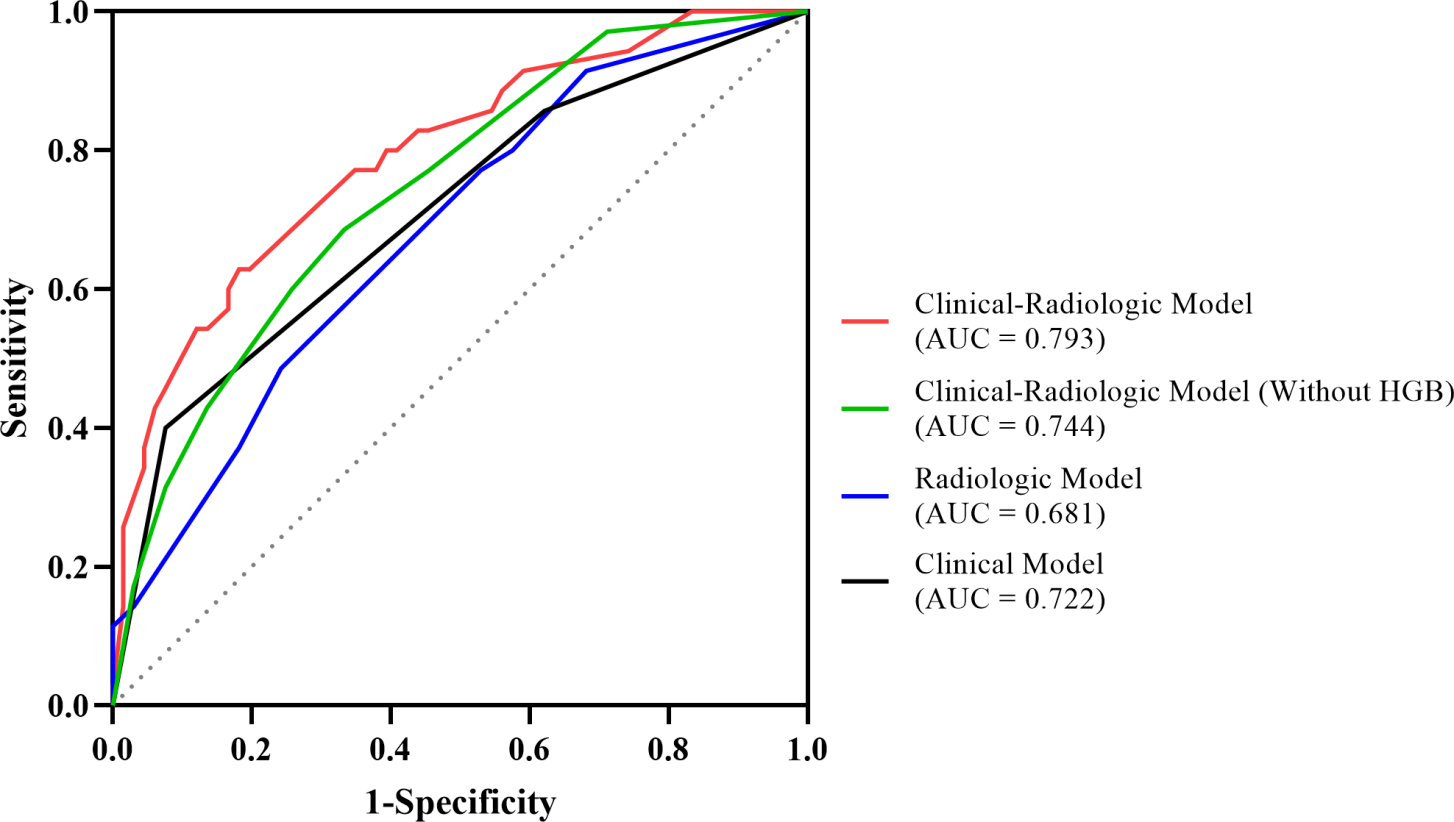
The ROC curves of the MTM-HCC prediction models. According to DeLong’s test, the clinical-radiologic model displayed better prediction performance than the radiologic model (*p* = .03) and the clinical model (*p* = .03).

The ROC curves demonstrated the best cutoff values of 228 ng/mL AFP and 130.5 g/L hemoglobin level, and univariate and multivariate logistic regression (**Table 4**) indicated that serum AFP ≥ 228 ng/mL (OR = 4.101; 95% CI: 1.523, 11.722; *p* = .006), hemoglobin ≥ 130 g/L (OR = 3.943; 95% CI: 1.466, 11.710; *p* = .009), tumors without enhancing capsules (OR = 3.274; 95% CI: 1.209, 9.755; *p* = .03) and intratumor hypoenhancement (OR = 2.724; 95% CI: 1.033, 7.467; *p* = .045) were independent predictors for MTM-HCCs. The predictive performance of each independent predictor and the three models (C model, R model, and CR model) are illustrated in **Table 5**. Intratumor hypoenhancement and tumors without an enhancing capsule were identified 57.1% (20/35) and 65.7% (23/35) of MTM-HCCs. The R model had a higher specificity of 75.8%, whereas the C model had a much higher specificity of 87.8% but an unsatisfactory sensitivity of 45.7%. In comparison, the CR predictive model demonstrated a specificity of 81.8% and a sensitivity of 62.9%, with an AUC of 0.793 (95% CI: 0.701, 0.886). The diagnostic performance of the CR predictive model was significantly improved compared to the R (*p* = .03) and C models (*p* = .03) according to DeLong’s tests, but there was no significant difference between the CR models with and without HGB (*p* = .18). The ROC curves of each model are displayed in **Figure 4**.

**Table 4:** Univariate and multivariate analyses of clinical and imaging features for MTM-HCCs.

	Univariate logistic regression		Multivariate logistic regression	
Variate	Odds Ratio	*p*-value	Odds Ratio	*p*-value
Sex (male)	0.929 (0.336, 2.567)	.89		
Age	0.976 (0.947, 1.005)	.11		
Tumor location	0.618 (0.231, 1.656)	.34		
Tumor size > 5 cm	1.412 (0.615, 3.240)	.42		
BCLC stage	3.323 (1.189, 9.284)	.02		.25
Cirrhosis	0.487 (0.206, 1.153)	.10		
HBV infection	0.486 (0.191, 1.235)	.13		
Serum AFP ≥ 228 ng/mL	3.933 (1.619, 9.553)	.02	4.101 (1.523, 11.722)	***.006***
HGB ≥ 130.5 g/L	3.069 (1.250, 7.540)	.01	3.943 (1.466, 11.710)	***.009***
Non-smooth tumor margin	2.279 (0.694, 7.491)	.18		
Non-rim APHE	1.144 (0.257, 5.095)	.86		
Washout	0.917 (0.233, 2.871)	.75		
Without enhancing capsule	2.164 (0.926, 5.058)	.08	3.274 (1.209, 9.755)	***.03***
Intratumor hypoenhancement	0.350 (0.150, 0.816)	.02	2.724 (1.033, 7.467)	***.045***
Fat in mass	0.523 (0.032, 8.625)	.65		
Blood products in mass	2.194 (0.236, 20.417)	.49		
Mosaic architecture	0.856 (0.363, 2.016)	.72		
Peritumoral hyperenhancement	0.395 (0.143, 1.090)	.07		.10
Internal artery	0.957 (0.418, 2.189)	.92		
Tumor in vein	0.475 (0.152, 1.484)	.20		

MTM, macrotrabecular-massive; AFP, alpha-fetoprotein, BCLC, Barcelona Clinic Liver Cancer; APHE, arterial phase hyperenhancement; HGB, hemoglobin.

Data in parentheses are 95% confidence intervals.

Significant values (p < 0.05) are presented in bold.

**Table 5:** Diagnosis performance of MTM-HCC predictive models.

Predicting Models	AUC	SPE	SEN	ACC	PPV	NPV
enhancing capsule	0.594 (0.478, 0.710)	53.0%	65.7%	57.5%	42.6%	74.5%
intratumor hypoenhancement	0.627 (0.511, 0.743)	68.2%	57.1%	64.4%	48.8%	75.0%
AFP ≥ 228 ng/mL	0.651 (0.535, 0.768)	78.8%	51.4%	69.3%	56.3%	75.4%
HGB ≥ 130.5 g/L	0.629 (0.516, 0.742)	51.5%	74.3%	59.4%	44.8%	79.1%
Radiologic (R) model	0.681 (0.575, 0.787)	75.8%	48.6%	66.3%	51.5%	73.5%
Clinical (C) model	0.722 (0.619, 0.825)	87.8%	45.7%	73.3%	66.7%	75.3%
Clinical-Radiologic (CR) model	0.793 (0.701, 0.886)	81.8%	62.9%	75.2%	64.7%	80.6%
Clinical-Radiologic (CR) model (without HGB)	0.744 (0.646, 0.841)	66.7%	68,6%	67.3%	52.2%	80.0%

MTM-HCC, macrotrabecular-massive hepatocellular carcinoma; AFP, alpha-fetoprotein; HGB, hemoglobin; SPE, specificity; SEN, sensitivity; ACC, accuracy; PPV, positive predictive value; NPV, negative predictive value; AUC, area under the ROC curve.

C model includes AFP and HGB.

R model includes enhancing capsule and intratumor hypoenhancement.

CR model includes AFP, HGB, enhancing capsule, and intratumor hypoenhancement.

Numbers in parenthesis are 95% confidence intervals.

### 3.4 Subgroup analysis

**Figure 5:**
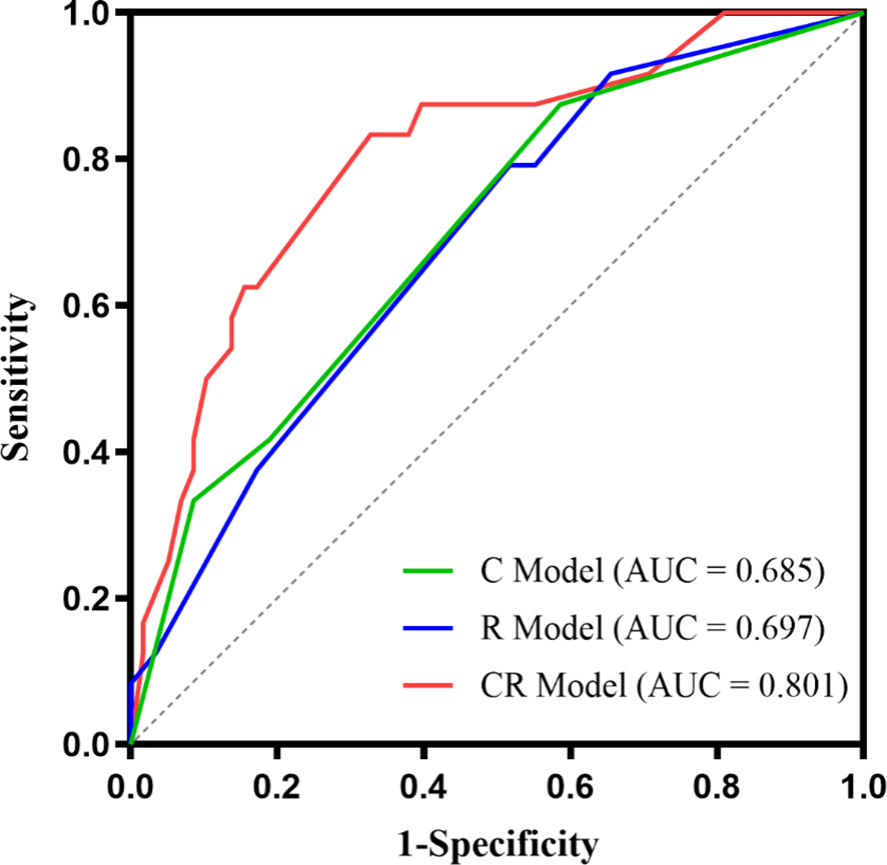
The ROC curves of the three models in BCLC 0-A stage subgroup.

The performance of the three models in BCLC 0-A subgroup are illustrated in **Table 6** and **Figure 5**. The AUC value of the CR model achieved 0.801 (95% CI: 0.695, 0.907), outperforming the C model with the value of 0.697 (95% CI: 0.580, 0.814) according to DeLong’s test (*p* = .01). Meanwhile, the AUC values between the CR model and the R model had no significant differences (*p* = .06).

**Table 6:** Diagnosis performance of MTM-HCC predictive models in BCLC 0-A subgroup.

Predicting Models	AUC	SPE	SEN	ACC	PPV	NPV
R model	0.685 (0.566, 0.805)	91.4%	33.3%	60.4%	61.5%	76.8%
C model	0.697 (0.580, 0.814)	79.3%	41.7%	55.4%	45.5%	76.7%
CR model	0.801 (0.695, 0.907)	91.4%	41.7%	62.4%	66.7%	79.1%

MTM-HCC, macrotrabecular-massive hepatocellular carcinoma; SPE, specificity; SEN, sensitivity; ACC, accuracy; PPV, positive predictive value; NPV, negative predictive value; AUC, area under the ROC curve.

C model includes AFP and HGB.

R model includes enhancing capsule and intratumor hypoenhancement.

CR model includes AFP, HGB, enhancing capsule, and intratumor hypoenhancement.

## 4 Discussion

The newly defined histological subtype MTM-HCC is associated with gene mutations, aggressive biological behavior, and poor prognosis regardless of surgical resection or radiofrequency ablation (4–6). In this study, we constructed predictive models for MTM-HCC based on clinical characteristics and CECT image findings, confirming high serum AFP, high hemoglobin, tumors without enhancing capsules, and intratumor hypoenhancement (for more than 20% of the whole tumor) were independent predictors for MTM-HCCs. The clinical-radiologic (CR) model, with an AUC value of 0.793, outperformed the radiologic (R) model and the clinical (C) model in terms of predicting MTM-HCCs. Additionally, the CR model demonstrated its ability to identify MTM-HCCs in patients with BCLC 0-A stage in the subgroup analysis.

The present study demonstrated that intratumor hypoenhancement and tumors without enhancing capsules were independent radiologic predictors of MTM-HCCs, which was consistent with previous studies (10–13, 15, 16). Intratumor hypoenhancement, including severe ischemic or necrosis, is related to endothelial-specific molecule 1 (ESM-1), angiopoietin 2 (Ang-2), and vascular endothelial growth factor A (VEGFA) expression in the tumor leading to peripheral tumor angiogenesis, hypoxia, rapid growth, and subsequent reduced central perfusion (25, 26). Moreover, necrosis correlates with poor survival in HCCs (27, 28), which could also explain the unsatisfying prognosis of MTM-HCCs. Tumors without enhancing capsules, as another independent radiologic predictor for MTM-HCC, are associated with aggressive biological behavior, which tends to have a higher incidence of direct liver invasion (29) or a strong correlation with *TP53* mutations (30), which might explain why MTM-HCCs more frequently invade adjacent liver parenchyma or vascular systems (6).

Alpha-fetoprotein (AFP) is a biomarker strongly associated with developing HCC and advanced stages (31). Our results depicted that higher serum AFP was also an independent predictor for MTM-HCC in line with previous studies (6, 10, 12, 32). Notably, higher hemoglobin level was another independent predictor of MTM-HCC. Considering the association between MTM-HCC and high expression of hypoxia-related genes like *EPO*, *CAIX*, and *VEGFA* (25), EPO might stimulate hemoglobin to produce compensatory oxygen for tumors. A similar phenomenon was reported by Emara et al., who observed increased hemoglobin in patients with glioma due to the hypoxic microenvironment (33). Calderaro et al. reported that MTM-HCC has a characteristic highly activated angiogenic microenvironment (25). In addition, Xue et al. found a higher level of hemoglobin in HCC related to tumor angiogenesis from Vasohibin 2 upregulation (34), which requires further investigation.

The present study found that the inclusion of the clinical characteristics to identify MTM-HCCs improved the predictive performance, but there was no significant difference between models within or without HGB, and the clinical-radiologic (CR) model has the highest AUC value. The predictive performance of our CR model is similar to previous studies, but our model had a higher AUC value and higher specificity than Feng’s model, which included AFP, tumor necrosis, and hemorrhage (12), possibly due to the contribution of lab test results. Additionally, our model had better sensitivity than several reported MRI-based predictive models (11, 13, 15). Furthermore, some features employed in previous studies, like tumor hemorrhage and intratumor fat, might lead to the relatively high specificity of the predictive model due to their rarity in the real clinical scenario. Our radiologic (R) model had an AUC value of only 0.681, with a specificity of 75.8% and a sensitivity of 48.6%. This was worse than our CR model, which had an AUC value of 0.793. A similar study by Liang et al. (15) also found low AUC values of 0.644-0.699 when combining any two imaging features of enhancing capsule, blood products in mass or ascites. However, Shan et al. (35) were able to construct a predictive model for MTM-HCC using aspartate aminotransferase, AFP, and prothrombin time, achieving a C-index of 0.723. Therefore, we believed that adding clinical characteristics can increase the robustness of the predictive model. Remarkably, our CR model was able to identify MTM-HCCs in early-stage patients (BCLC 0-A stage). This result could significantly influence therapy decisions for patients with this subtype, promoting more aggressive interventions such as wider tumor resection margins, more intensive follow-up schedules, and a more comprehensive selection of adjuvant therapies, ultimately improving clinical outcomes.

Previous studies have demonstrated that MTM-HCCs tend to be larger than non-MTM-HCCs (6, 10, 11, 36). Although a larger tumor size of HCCs correlates with a higher histologic grade, vascular invasion, tumor recurrence, and extrahepatic metastasis (37–39), there was no significant difference in tumor size or the long-to-short axis ratio between MTM-HCCs and non-MTM-HCCs in our study, in contrast to previous findings. This difference in our study might be due to the small sample size and strict inclusion criteria. All the HCCs in our study were confirmed pathologically, leading to exclusion of smaller HCC lesions that underwent ablation other than hepatectomy.

Our study has several limitations that should be acknowledged. Firstly, being a single-center retrospective study, selection bias may have affected the results. Secondly, due to the lack of prognosis information and validation cohort, future studies with a larger multicenter cohort and including prognosis information are necessary to validate our findings. Additionally, future quantitative studies using radiomics or deep learning methods may provide high-throughput data to construct a more robust predictive model.

In conclusion, a prediction model combining imaging features and clinical data can identify MTM-HCCs even in early-stage patients and has better predictive performance than imaging features or clinical data alone.

## Data availability statement

The original contributions presented in the study are included in the article/supplementary material. Further inquiries can be directed to the corresponding authors.

## Ethics statement

The studies involving human participants were reviewed and approved by the Institutional Review Board of the Second Affiliated Hospital of South China University of Technology. Written informed consent for participation was not required for this study in accordance with the national legislation and the institutional requirements.

## Author contributions

CH and WZ contributed equally to this work and share the first authorship. RY and XJ contributed equally to this work and CH, WZ, and RY conducted the literature search. CH, WZ, XW, RY, and XJ designed the study. JL, WY, YW, NW, and WD collected the data. CH, WZ, YZ, JL, and RY analyzed the data. All authors verified the data. CH, WZ, RY, and XJ edited the manuscript. RY, XW, and XJ reviewed the manuscript. All authors contributed to the article and approved the submitted version.
